# Intracranial Microhemorrhages in a Patient With Tubercular Meningitis (TBM): A Case Report

**DOI:** 10.7759/cureus.41462

**Published:** 2023-07-06

**Authors:** Shubhajeet Roy, Shikhar S Gupta, Syed N Muzaffar

**Affiliations:** 1 Faculty of Medical Sciences, King George's Medical University, Lucknow, IND; 2 Critical Care Medicine, King George's Medical University, Lucknow, IND

**Keywords:** septic shock, pyrexia of unknown origin (puo), tubercular meningitis (tbm), intra-cranial microhemorrhages, intracranial tuberculosis

## Abstract

Intracranial tuberculosis (TB) is the most serious form of systemic TB and constitutes an important cause of morbidity and mortality in underdeveloped countries. Central nervous system TB is a difficult diagnosis to make, and treat, especially in the developing nations. Intracranial hemorrhage is one of the rare complications of intracranial TB. We are reporting a case of a 70-year-old male patient who presented to the neurology ward with complaints of persistent high-grade fever associated with significant weight loss, night sweats, and hemolysis for two months. Cerebrospinal fluid analysis was suggestive of tubercular meningitis. He was started on first-line antitubercular therapy. After two weeks, he developed respiratory distress, and invasive mechanical ventilation was started. He was then referred to the Intensive Care Unit of the Critical Care Medicine department. Susceptibility weighted images magnetic resonance imaging (MRI) revealed multiple nodular and ring-enhancing lesions with multifocal areas of microhemorrhages in the brain parenchyma, and leptomeningeal enhancement in bilateral sylvian, perimesencephalic, prepontine and cerebellopontine angles. A tracheostomy was performed. He also developed septic shock for 72 hours, secondary to Pseudomonas aeruginosa and Acinetobacter baumannii ventilator-associated pneumonia, and Klebsiella bacteremia for which intravenous noradrenalin, Carbapenem and Colistin were administered. The patient improved within eight weeks. Our case presented with altered sensorium for the past three to four days but generally, there are other common features like headache, seizures, focal neurological deficit, and raised intracranial pressure. MRI findings of caseating tuberculomas reveal isointense to hypointense signals on both T2 and T1 weighted images with ring enhancement, which are in resemblance with the MRI findings of our case.

## Introduction

Tuberculosis (TB) has been one of the most frequently encountered diseases in the world for which the medical fraternity has dedicated years to decrease its outcome but still a large chunk of the population remains under its clutches. Pulmonary TB is the most commonly encountered manifestation, however extrapulmonary TB, especially Central Nervous System (CNS) involvement occurs in 2-5% of patients with tuberculosis (TB), and up to 10% of patients with immunocompromised conditions [1]. Intracranial TB is commonly caused by hematogenous spread of Mycobacterium tuberculosis bacilli, due to erosion of an affected lymph node into nearby vessels. The disease focus is commonly found in the lung or gastrointestinal tract [1-3]. CNS TB as an entity is difficult to diagnose and treat in developing countries, hence it is a major cause of morbidity and mortality. Intracranial hemorrhage is an extremely rare complication where cases of it have been reported in cerebral lobes, cerebellum, basal ganglia, ventricles, and suprasellar region [4]. In this study, we report a case of a 70-year-old male patient who got referred to our ICU with a diagnosis of tubercular meningitis (TBM). MRI brain revealed intra-cranial microhemorrhages in this patient, which have not been commonly reported in intracranial TB.

## Case presentation

A 70-year-old male patient was apparently asymptomatic two months back, when he presented with complaints of persistent high-grade fever associated with significant weight loss, night sweats, and hemoptysis. His past medical history included hypertension for 15 years, reactive airway disease for 15 years, and pleural TB 20 years back. He was referred to our hospital from a rural health care centre, because of altered sensorium for the last three to four days, for which he was admitted to the department of neurology, and a working provisional diagnosis of pyrexia of unknown origin (PUO), along with altered sensorium, was made. His past medication history included angiotensin receptor blockers and calcium channel blockers for his hypertension, bronchodilators for his respiratory complaints, and antitubercular therapy (ATT) for six months (Isoniazid, Rifampicin, and Ethambutol for six months, and Pyrazinamide for two months) 20 years back. During his stay on the ward, the best Glasgow Coma Scale (GCS) score recorded was E2V3M4. Non-contrast computed tomography (NCCT) of the head was done on admission, which did not show any abnormality. The patient underwent lumbar puncture and cerebrospinal fluid (CSF) analysis showed TBM, with a CSF pressure of 375 mm of water (reference: 50-180 mm of water), total leukocyte count (TLC) being 910/mm^3^ (reference: <5 leukocytes), differential leukocyte count (DLC) showing lymphocytes at 80%, polymorphs at 20%, CSF proteins being 275 mg/dL (reference: 15-45 mg/dL), CSF lactate 7.2 mmol/L (reference: 1.1-2.4 mmol/L) and CSF/serum glucose ratio being 0.51 (reference: >0.6). Serum hemoglobin was 13.2 g/dL (reference: 14-18 g/dL), red blood cell count was 5.2 million/cu.mm (reference: 4.0-5.9 million/cu.mm), total leukocyte count was 13,000/cu.mm (reference: 4,000-11,000/cu.mm), differential leukocyte count showed 66% neutrophils (reference: 55-70%), and 31% lymphocytes (reference: 20-40%), and platelet count was 3.3 x 10^5^/mcL (reference: 1.5-4.5 x 10^5^/mcL). Disseminated intravascular coagulation (DIC) profile, liver and renal function tests were within normal limits. C-reactive protein levels were 42 mg/L (reference: <30 mg/L). Serum adenosine deaminase (ADA) levels were found to be 26 IU/L (reference: 40 IU/L), serum proteins were 700 mg/dL (reference: 6,000-8,000 mg/dL), serum procalcitonin was 0.9 ng/mL (reference: <0.1 ng/mL), and fasting blood glucose levels were 37 mg/dL (reference: 70-100 mg/dL). Tests for cytoplasmic anti-neutrophil cytoplasmic antibodies (C-ANCA) and perinuclear anti-neutrophil cytoplasmic antibodies (P-ANCA) yielded negative results. CSF workup for fungus, malignant cells and gram stain was negative. GeneXpert, Bactec Mycobacteria Growth Indicator Tube (MGIT), and acid-fast bacilli test by Ziehl-Neelsen staining showed the presence of drug-sensitive (first line drugs- Isoniazid, Rifampicin, Pyrazinamide and Ethambutol) Mycobacterium tuberculosis bacilli. Screening for sepsis and workup for tropical illness were all within normal limits. Chest X-ray showed normal findings, and digital subtraction angiography for head and neck vessels showed no abnormalities or malformations. ATT (isoniazid, rifampicin, pyrazinamide, ethambutol, and streptomycin) was initiated along with oral steroids. Preparation was kept for intubation with all measures to prevent aspiration. Meanwhile, correction for hyponatremia was being continued and any recovery in sensorium was being monitored. The patient was on small trophic enteral feeds with regular monitoring of gastric residual volumes (GRVs) along with supplemental parenteral nutrition. Reaction in ventilator-associated pneumonia (VAP) bundle, hand hygiene, head of bed elevation, oral care with chlorhexidine, stress ulcer prophylaxis and deep vein thrombosis (DVT) prophylaxis were being administered. After two weeks of stay in the ward, he had respiratory distress due to aspiration pneumonitis, for which invasive mechanical ventilation was initiated. He was then referred to the intensive care unit (ICU) of Critical Care Medicine department with a diagnosis of tubercular meningitis (TBM), along with aspiration pneumonia and type I respiratory failure.

**Figure 1:**
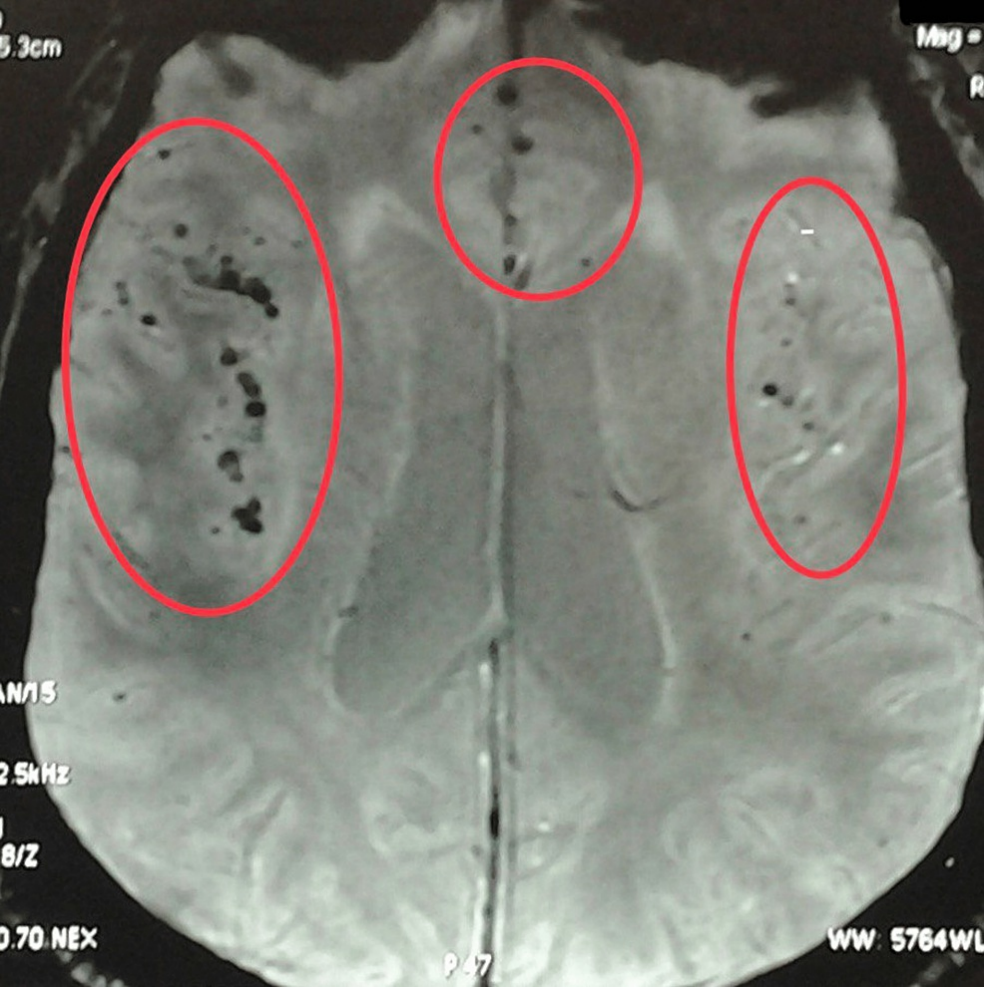
Susceptibility weighted magnetic resonance imaging of the brain showing microhemorrhages in bilateral frontoparietal regions (red circles).

On ICU admission, he was sedated and his Richmond Agitation Sedation Score (RASS) was -3 to -4. His GCS was E2VTM3, the pupils were bilaterally 3 mm and reacted briskly to light, the plantars showed bilateral normal reflex, heart rate was 75/min, blood pressure was 140/90 mm of Hg (without any vasopressor support), warm peripheries, and was afebrile to touch. During his ICU stay, he had waxing and waning sensorium. His Modified Rankin Score was zero. His liver function test (LFT) was within normal limits. The patient was resuscitated by stabilization of airway, breathing and circulation. Fluid resuscitation was done with small fluid challenges along with screening 2D-echo (for inferior vena cava size and variation and left ventricular contractility) and lung ultrasonography to ensure judicious fluid resuscitation. Possible differential diagnoses considered for the neurological deterioration were structural CNS causes (stroke, intracranial hypertension), metabolic causes and non-convulsive seizure activity. So, T1, T2, T2 flair, diffusion-weighted images (DWI) and susceptibility-weighted images (SWI) magnetic resonance imaging (MRI) of the brain was obtained, which revealed multiple nodular and ring-enhancing lesions with multifocal areas of blooming (microhemorrhages) in the brain parenchyma, and leptomeningeal enhancement in bilateral sylvian, perimesencephalic, prepontine and cerebellopontine angles suggestive of TBM (Figure 1).

ATT was continued. The steroids were also continued for around a month and then tapered off. CSF culture reported positive for tuberculosis after eight weeks of incubation in Löwenstein-Jensen medium. In anticipation of prolonged mechanical ventilation, a tracheostomy was done. During the intercurrent issues, he had developed septic shock for 72 hours, secondary to Pseudomonas aeruginosa and Acinetobacter baumannii ventilator-associated pneumonia (VAP, which was diagnosed on the basis of Clinical Pulmonary Infectious Score (CPIS) criteria inclusive of temperature, tracheal secretions, total leukocyte count, oxygenation, chest X-ray and microbiology of tracheal secretions. Susceptibility pattern: only sensitive to colistin), and Klebsiella bacteremia for which vasopressor support in the form of intravenous (IV) noradrenaline 0.5-1 mcg/kg/min was administered, and culture-directed antibiotic therapy was given, in the form of intravenous Carbapenem with Colistin for two weeks. Over a period of eight weeks, his sensorium improved to E4VTM6, and gradually, he was liberated from mechanical ventilation. Decannulation could not be done due to neuromuscular weakness. Eventually, he was discharged with a tracheostomy tube in situ, on room air, with a GCS of E4VTM6. The patient was advised anti-tubercular therapy to be continued, oral dexamethasone (as per the scheduled regimen for TBM), multivitamins, and calcium tablets during discharge.

## Discussion

CNS involvement in tuberculosis is often underdiagnosed in the earlier stages, since many diseases of CNS present with the same features, due to which clinicians often do not consider TB as a differential, which tends to be the most serious form, with high mortality. Our case is of a 70-year-old man, but CNS TB occurs most commonly (60-70%) in less than 20 years [4]. The manifestations of intracranial TB are secondary to parenchymal or meningeal involvement. The parenchymal disease commonly presents as solitary or multiple tuberculomas [1-2]. Intraparenchymal tuberculomas occur secondary to a focus of infection, elsewhere in the body. In adults, they commonly occur in the frontal or the parietal lobes. Intraventricular tuberculomas, manifesting as hydrocephalus, meningitis, and ependymitis, have also been reported. Parenchymal involvement may also present as tuberculous abscesses, cerebritis, and tuberculous encephalopathy [3-7]. Tuberculomas, when formed, are avascular granulomatous formations consisting of mixed epithelioid and giant cells with lymphocytic predominance around a central zone of caseous necrosis, and bleeding from them is rare since TBM is known to involve surrounding vessels and cause vasculitis which can lead to aneurysms and venulitis that can rupture and lead to intracranial hemorrhages [4,8-10]. Tubercular abscesses are found in less than 10% of cases and are more common in elderly patients or those with compromised immunity.

TBM is caused by CSF seeding from rupture of the pial or subependymal granuloma. The involvement of a vessel in subarachnoid space may also cause the subsequent involvement of surrounding meninges [6-7]. Rarely, meningeal involvement may be due to the spread from mastoid or sphenoid sinuses. Meningeal enhancement is the most sensitive feature for identifying TBM. The most common complication of TBM is communicating hydrocephalus which occurs secondary to obstruction of CSF flow by meningeal exudates in the basal cisterns. In some cases, hydrocephalus may be non-communicating as a result of obstruction from tuberculomas or tuberculous abscesses. The combination of basal exudates, infarcts, and hydrocephalus is considered the diagnostic triad of TBM. Cranial nerve palsies can also occur in TBM due to vascular compromise or due to entrapment of nerve in basal exudates and it most commonly affects the third, fourth, and sixth cranial nerves [7].

Our case was referred to with the diagnosis of tubercular meningitis with altered sensorium for the past three to four days whereas usually, there are other common presenting features including headache, seizures, focal neurological deficit, and raised intracranial pressure [4]. On MRI, caseating tuberculomas demonstrate isointense to hypointense signals on both T2 and T1 weighted images with ring enhancement, which are in concordance with the MRI findings of our case [11].

Intracranial hemorrhage is a rarely reported entity with only a few case reports mentioning complications like intraventricular, intracerebral, and subarachnoid hemorrhages in such patients [8-11,12-17]. It may be an additional cause of morbidity and mortality. The occurrence of microhemorrhages could be attributed to the disintegration of the internal elastic lamina of vessel walls secondary to chronic granulomatous inflammation or due to the rupture of mycotic aneurysms [15]. In most of these reports, the outcome has been poor.

## Conclusions

Intracranial haemorrhage may be an atypical presentation of intracranial TB, which may influence the prognosis of the disease and henceforth should be kept in mind while treating patients of intracranial TB. Our patient had presented with altered mentation, whereas there are other presenting features, which are commonly encountered, including headache, seizures, focal neurological deficit, and raised intracranial pressure, which was not present in our patient. Hence a high index of suspicion should be borne while managing patients with such symptoms. Early diagnosis and prompt treatment can help in bringing down the mortality rate of such patients, given the fact that already CNS TB has a high mortality rate worldwide.
